# Inherited genetic predispositions in *F13A1* and *F13B* genes predict abdominal adhesion formation: identification of gender prognostic indicators

**DOI:** 10.1038/s41598-018-35185-x

**Published:** 2018-11-16

**Authors:** Donato Gemmati, Savino Occhionorelli, Veronica Tisato, Marco Vigliano, Giovanna Longo, Arianna Gonelli, Maria G. Sibilla, Maria L. Serino, Paolo Zamboni

**Affiliations:** 1 Department of Biomedical & Specialty Surgical Sciences and Centre Haemostasis & Thrombosis, Section of Medical Biochemistry, Molecular Biology & Genetics, University of Ferrara, corso Giovecca 203, 44121 Ferrara, Italy; 2 Department of Morphology, Surgery & Experimental Medicine, University of Ferrara and Vascular Diseases Centre, Unit of Translational Surgery, University-Hospital of Ferrara, via Aldo Moro 8, 44124 Cona-Ferrara, Italy; 3 Department of Morphology, Surgery & Experimental Medicine and LTTA Centre, University of Ferrara, via Fossato di Mortara 70, 44121 Ferrara, Italy; 4 University Center for Studies on Gender Medicine, University of Ferrara, 44121 Ferrara, Italy

## Abstract

Abdominal adhesions (AA) account for the most common complication of peritoneal surgery with bowel obstruction being the severest problem in the absence of effective predicting biomarkers. Anti-AA-barriers or adhesiolysis did not completely prevent bowel obstruction, although there is evidence they might reduce related complications requiring reoperation. In addition, gender-related predispositions have not been adequately investigated. We explored the role of coagulation Factor XIII (*F13A1* and *F13B* subunit-genes) in patients following laparotomy, mostly median/lower median incision line. Globally, 426 patients (54%,♀), were PCR-SNP-genotyped for FXIIIA V34L (rs5985), FXIIIA P564L (rs5982), FXIIIA Y204F (rs3024477) and FXIIIB H95R (rs6003). Patients’ clinical phenotypes were: Group-A (n = 212), those who developed AA, and 55.2% of them developed bowel obstruction (subgroup-A1), the remaining were subgroup-A2; Group B (n = 214) were those who did not develop AA (subgroup-B1; 53.3%) or symptoms/complications (subgroup-B2). Among different laparotomy, colon surgery associated with AA at a major extent (OR = 5.1; 3.24–7.8; P < 0.0001) with different gender scores (♀OR = 5.33; 2.32–12.23; P < 0.0001 and ♂OR = 3.44; 1.58–7.49; P < 0.0001). Among SNPs, P564L (OR = 4.42; 1.45–13.4; P = 0.008) and Y204F (OR = 7.78; 1.62–37.3; P = 0.01) significantly predicted bowel obstruction and survival-analyses yielded interesting gender distinctions (♀HR = 5.28; 2.36–11.8; P = 0.00005; ♂HR = 2.22; 1.31–3.85; P = 0.0034). Active compounds preventing AA belong to the anticoagulant/fibrinolysis areas, suggesting them candidate investigation targets. We identified novel prognostic markers to predict AA/bowel obstruction giving insights to design novel therapeutic and gender prevention programs.

## Introduction

Abdominal adhesions (AA) are considered a major surgical issue even when the primary surgical intervention concludes without apparent complications for either open or laparoscopic procedure (LAP)^1,2^. AA are widely recognized as one of the most common causes of complications after surgery mainly including bowel obstruction, female infertility, difficulty of reoperation, and chronic pain^2^. The incidence of AA ranges from 37% to 90% of the global surgical interventions and despite aetiology and pathophysiology have been widely investigated, effective preventive interventions or recognition of predisposing factors are still missing, leaving their occurrence substantially unaffected^3,4^.

**Figure 1:**
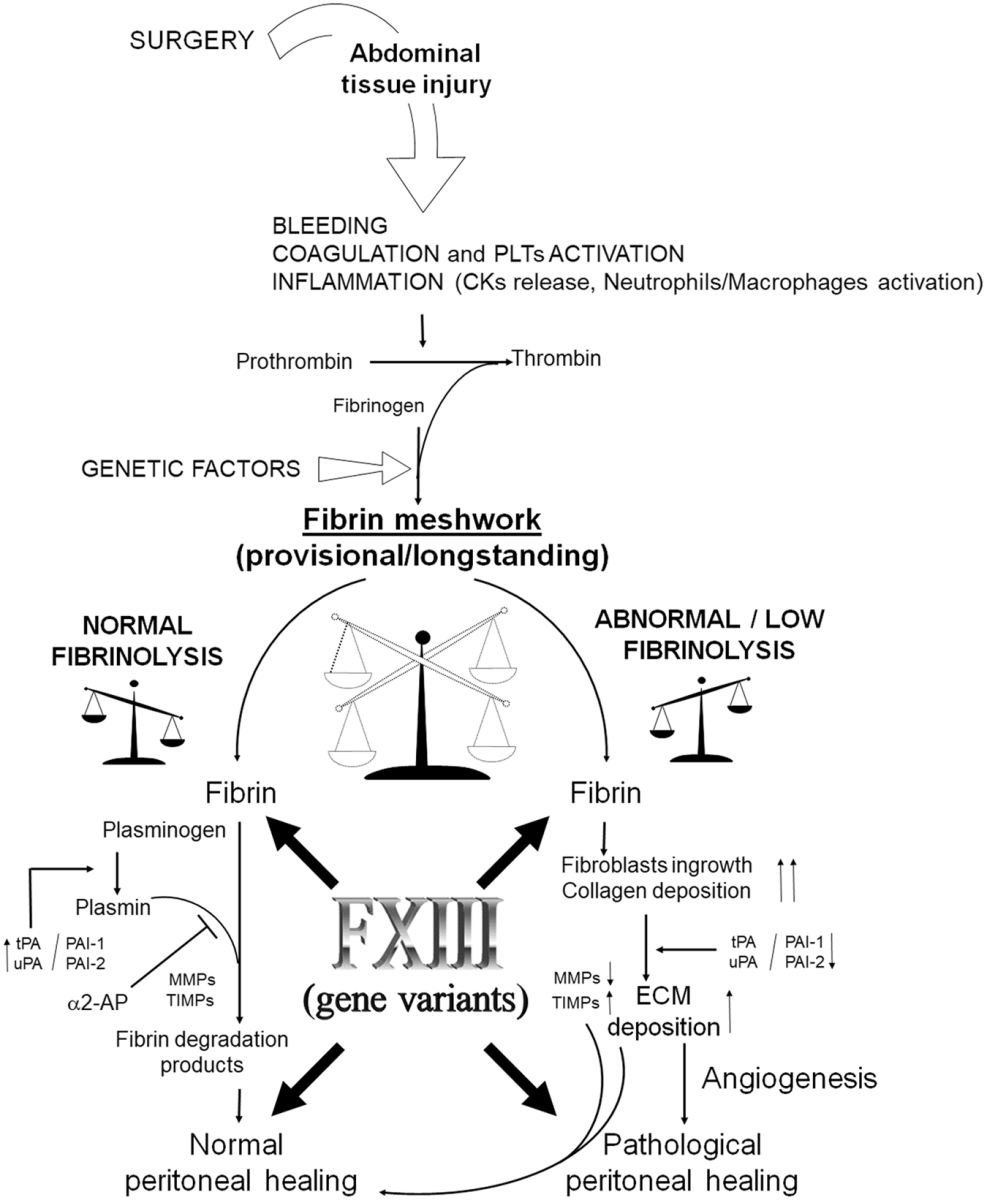
Schematic representation of the cross-talk between blood coagulation and inflammation involved in the normal Fibrin matrix resolution or development of abdominal adhesions. CKs, Cytokines; PLTs, Platelets; tPA, tissue plasminogen activator; uPA, urokinase-type plasminogen activator; PAI-1 and PAI-2, plasminogen activator inhibitors 1 and 2; FXIII, Factor XIII (A2B2); α2-AP, Alpha 2-antiplasmin; MMPs, Matrix Metalloproteinases; TIMPs, Tissue Metalloproteinase Inhibitors.

The main mechanisms responsible for adhesion formation should be investigated within those belonging to the wound healing and reparative processes^5^. After surgery, a fine and complex sequence of reactions involving soluble mediators (e.g. inflammatory cytokines/growth factors) and different cell subsets including neutrophils, macrophages as well as fibroblasts and resident mesenchymal stem cells take place during normal peritoneal repair. Any unbalance or disturbance in inflammatory and cell-migration processes, in the blood coagulation and fibrinolysis systems, or alterations in matrix deposition and remodelling phases, may also lead to AA formation^6–10^. Noteworthy, blood coagulation and inflammatory processes are mutually connected both at the molecular and patho-physiological levels, showing a cross-talk between key players^3^, including Fibrinogen, Factor XIII (FXIII), Thrombin, Plasminogen, Metalloproteases (MMPs) and inflammatory cytokines, *in primis* TNFα and different ILs, that synergize in AA establishment (Fig. 1). In addition, gender, age, hormones and genetics by impacting on inflammation, coagulation and fibrinolysis, may modify the risk of AA occurrence^4^. After surgery, the injured tissue starts a complex series of processes aimed at repairing and replacing the lesion with scar tissue to restore the normal physiological and mechanical features. The formation of 3D-fibrin meshwork provides the first provisional scaffold for cell recruiting, spreading and neo-vessels formation before deposition of collagen and fibronectin, the key components of definitive extracellular matrix (ECM)^11^. FXIII and Fibrinogen are the two coagulation factors mainly responsible for the formation of the provisional elastic 3D-fibrin meshwork contributing to the normal and balanced healing^12^.

Fibrin meshwork should be transitory and stay for a limited time during adequate and physiological healing; afterward it should be degraded by the endogenous fibrinolytic system while restoring the proper tissue structure and function. When these processes are unrestrained and deregulated, they may lead to the generation of stiff scrapes characterized by abnormal cellular component and higher fibrotic constituent, resulting in a long-lasting or permanent non-elastic tissue. Although these molecular mechanisms take place in the early reparative phases simply affecting the 3D-fibrin meshwork, adhesions continue to evolve during time and their clinical manifestations may occur several years after surgery^4^. In this light, we aimed to investigate the role of FXIII in AA formation, being one of the key factors involved in the covalent cross-linking of both provisional and definitive ECM components^12^.

Factor XIII is a multitasking protein at the intersection of coagulation, fibrinolysis and inflammation^12–14^. The zymogen circulates in blood as a pro-transglutaminase complex of two enzymatic A-subunits, and two carrier B-subunits (FXIIIA2B2) that prevent the early and wasteful activation/degradation of the active A-subunits^12^. In the classical field of coagulation, FXIII cross-links fibrin fibres, supporting platelets adhesion to the damaged endothelium and allowing the maintenance of a solid and elastic architecture. Moreover, in the last decades, FXIII has raised growing attention in several fields including tissue healing, angiogenesis, tissue repairing and regenerative medicine^12–15^.

Our group has been working for a long time to disclose the role of FXIII both in *in vitro* models and *in vivo* to define the impact of its residual circulating levels in different pathological contexts^12,16–20^. Moreover, we demonstrated that common genetic variants (i.e. single nucleotides polymorphisms, SNPs) within the genes of FXIII A and B subunits (*F13A1* and *F13B*), show a great pharmacogenetics role in chronic and post-surgical lesions, strongly influencing prognosis and healing time as well as the size of the lesions, when considered alone or in combination with other molecular markers^21–24^. The mechanistic explanation of the connections between *F13A1* or *F13B* SNPs and other genes/molecular markers in the context of wound healing has been addressed by our group, leading to the proposal of a model in reparative processes after tissue injury with strong implications in molecular medicine^25^.

Few studies investigated individual predispositions to post-operative AA; among these it has been reported the role of one SNP within the pro-inflammatory cytokine IL-1RA (IL-1 receptor antagonist) in women^26^ and the relationships between AA, intestinal obstruction and HLA subtype in children^27^. Additionally, the coagulation and fibrinolytic balance has been found to be strongly affected by genetic variants, though AA-association studies have not been widely performed^4^. Finally, different expression profiles of specific genes between fibroblasts taken from adhesions and normal human peritoneum confirm the key patho-physiological role of gene polymorphisms^28,29^. Overall, the high degree of genetic control of the coagulation-fibrinolysis balance makes them candidate-players in AA pathogenesis.

## Results

### Study design and population characteristics

**Figure 2:**
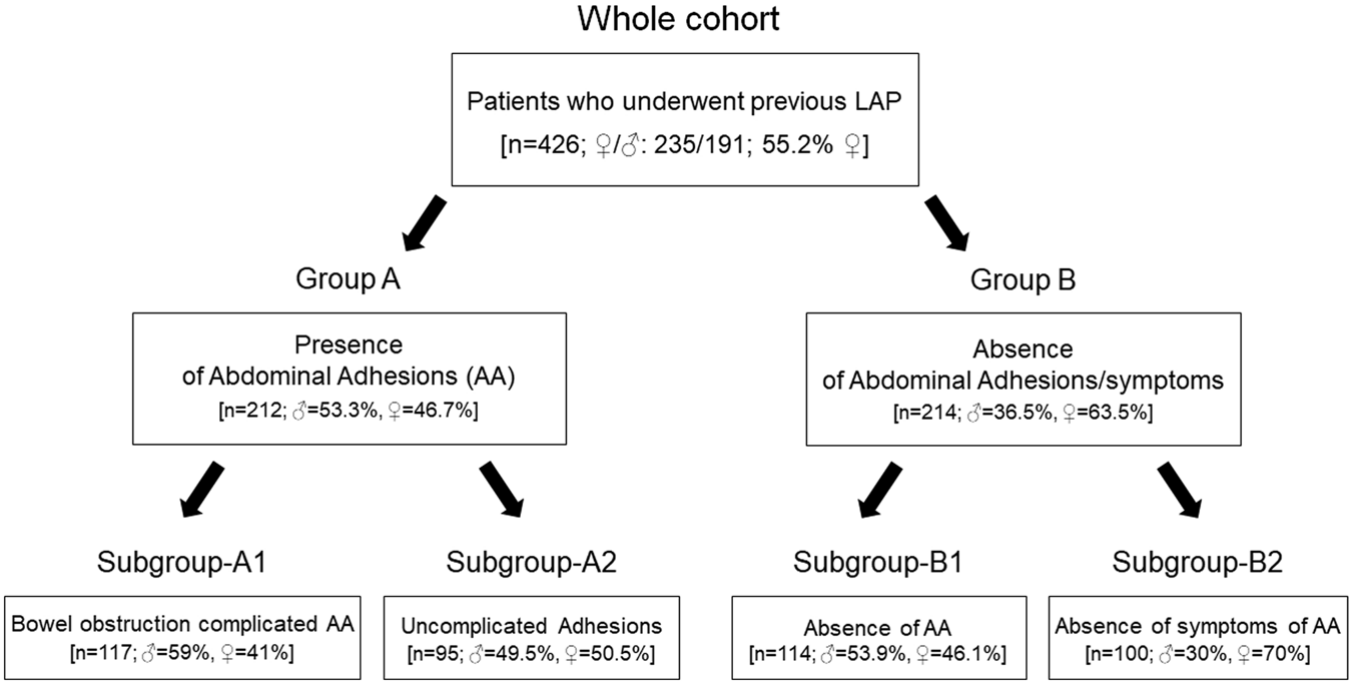
Flowchart of the study. The picture shows the whole group of enrolled patients who underwent to a previous LAP surgical procedure and sub-group stratification according to the clinical phenotype.

Figure 2 shows the whole group of enrolled patients who underwent a previous LAP surgical procedure. Group-A included patients who developed bowel obstruction (subgroup-A1) and patients showing uncomplicated AA occasionally detected during a second LAP (subgroup-A2). Group-B included patients without AA occasionally confirmed during a second LAP (subgroup-B1) and patients who did not have symptoms indicative of AA (subgroup-B2).

Although in the whole group of patients females were slightly over-represented (55.2%), they were significantly under-represented among those who experienced AA (46.7% *vs* 63.5% in group A and group B respectively; P = 0.0005).

Table 1 shows the main clinical and epidemiological features of the patients’ population and the main types of the original LAP performed. The group presenting AA (group-A) showed a higher mean age (P = 0.007), a higher number of colon surgery interventions (P = 0.0001) and a lower number of appendectomy (P = 0.0001) as well other surgery (P = 0.029) than the control group-B. In our cohort of patients, males seemed more frequently to develop AA (group-A). In particular, males with previous colon surgery or appendectomy did not reach significant overrepresentation against females whilst males with previous cholecystectomy or splenectomy (other surgery) significantly exceeded females (P = 0.04). Finally, females with previous hysterectomy doubled among subgroup-B than among subgroup-A, though at a not significant extent.

**Table 1:** Clinical and epidemiological characteristics of enrolled cases stratified by gender.

Characteristics	Whole group (n = 426) [♀/♂, 235/191; 55.2% ♀]		P
	Group A (n = 212)	Group B (n = 214)	
Female sex (n; %)	99 (46.7)	136 (63.5)	**0.0005**
Age, years (mean ± SD)	69.76 ± 14.0	65.8 ± 15.5	**0.007**
**Original LAP (n)**			
*colon surgery (n* = *151)* ♀, n = 67 (%) ♂, n = 84 (%)	112 (74.2)	39 (25.8)	**0.0001**
	47 (70.1) 65 (77.4)	20 (29.8) 19 (22.6)	0.313
*appendectomy (n* = *123)* ♀, n = 72 (%) ♂, n = 51 (%)	43 (34.9)	80 (65.0)	**0.0001**
	22 (30.5) 21 (41.2)	50 (69.4) 30 (58.8)	0.223
*other surgery: splenectomy*, *cholecystectomy (n* = *93)* ♀, n = 37 (%) ♂, n = 56 (%)	37 (39.8)	56 (60.2)	**0.029**
	10 (27.0) 27 (48.2)	27 (73.0) 29 (51.8)	**0.040**
hysterectomy*, *n* = *59* (%)	20 (33.9)	39 (66.1)	0.13

Significant P-values are shown in bold. *Only females were computed in the analysis.

Table 2 compares those patients with AA who developed bowel obstruction after previous LAP (subgroup-A1) *vs* those with uncomplicated AA (subgroup-A2) and patients who did not develop AA after previous LAP (subgroup-B1) *vs* cases without evidence of symptoms related to AA (subgroup-B2).

**Table 2:** Clinical and epidemiological characteristics in the subgroups of enrolled cases stratified by gender.

Characteristics	Whole group (n = 426) [♀/♂, 235/191; 55.2% ♀]				P A1 vs A2	P B1 vs B2
	Group A (n = 212)		Group B (n = 214)			
	A1 (n = 117)	A2 (n = 95)	B1 (n = 114)	B2 (n = 100)		
Female sex (n; %)	49 (41.9)	50 (52.6)	65 (57.0)	71 (71)	n.s.	**0**.**034**
Age, years (mean ± SD)	68.9 ± 15.3	70.8 ± 12.2	68.7 ± 13.9	62.5 ± 16.5	n.s.	**0**.**002**
**Original LAP**						
*colon surgery (n = 151)* ♀, n = 67 (%) ♂, n = 84 (%)	69 (59)	43 (45.2)	28 (24.5)	11 (11)	<**0**.**0001**	
	28 (41.8) 41 (48.8)	19 (28.3) 24 (28.6)	13 (19.4) 15 (17.8)	7 (10.4) 4 (4.8)	n.s.	n.s.
*appendectomy (n = 123)* ♀, n = 72 (%) ♂, n = 51 (%)	22 (18.8)	21 (22.2)	42 (36.8)	38 (38)	**0**.**0014**	
	7 (9.7) 15 (29.4)	15 (20.8) 6 (11.8)	24 (33.3) 18 (35.3)	26 (36.1) 12 (23.5)	**0**.**01**	n.s.
*other surgery: splenectomy*, *cholecystectomy (n* = *93)* ♀, n = 37 (%) ♂, n = 56 (%)	15 (12.8)	22 (23.2)	29 (25.4)	27 (27)	**0**.**044**	
	3 (8.1) 12 (21.4)	7 (18.9) 15 (26.8)	13 (35.1) 16 (28.6)	14 (37.8) 13 (23.2)	n.s.	n.s.
hysterectomy*, n = 59 (%)	11 (18.6)	9 (15.2)	15 (25.4)	24 (40.8)	n.s.	n.s.
Years from first and second LAP (mean ± SD)	5.9 ± 8.5	9.66 ± 9.5	n.a.	n.a.	**0**.**025**	—

Significant P-values are shown in bold. *Only females were computed in the analysis.

Females were significantly over-represented in subgroup-B2 compared to B1 (P = 0.034), and B2 had a lower mean age (P = 0.002) when compared to B1. Conversely, no sex or age differences were observed between A1 and A2 subgroups.

Colon surgery was over-represented in subgroup-A1 with respect to the remaining A2, B1, B2 subgroups (P < 0.0001) with no difference in a sex-stratified sub-analysis. This suggests a stronger association of colon surgery with AA occurrence, regardless sex difference. Conversely, appendectomy was over-represented in subgroups-B1 and B2 (P = 0.0014), with significant differences in a sex-stratified sub-analysis particularly evident by comparing A1 *vs* A2 (P = 0.01). Cholecystectomy and splenectomy were equally distributed among subgroups, and though a slight under-representation among subgroup-A1 (P = 0.044), no significant sex-difference was observed. Finally, hysterectomy did not yield significant differences neither between A1 *vs* A2 nor between B1 *vs* B2 comparisons.

Of note, the mean time between the first LAP and the evidence of AA was significantly shorter in those who experienced bowel obstruction (subgroup-A1) compared with those (subgroup-A2) in which AA were accidentally revealed during a second independent surgery (5.9 ± 8.5 years *vs* 9.66 ± 9.5 years respectively; P = 0.025) thought in both groups AA existence might precede bowel obstruction or surgery respectively.

### Risk of developing AA or bowel obstruction according to different types of LAP

Table 3 shows the risk of developing AA (group-A) or related bowel obstruction (subgroup-A1) according to the different type of primary LAP performed. Colon surgery yielded the highest rate to develop AA in the whole group, almost 75% of cases who underwent to the intervention developed AA resulting in quite 5.0-folds increasing risk, with females showing slightly higher scores than males. Appendectomy and any type of the remaining LAPs considered (cholecystectomy or splenectomy or hysterectomy), did not show elevated risk for AA, and this was evident because no odds yielded rates above the unit-value in the whole comparison (groups A vs B), in the sub-analysis (subgroups A1 vs B1) or in the sex sub-setting. Interestingly, by comparing only those cases that developed bowel obstruction (subgroup-A1) with those who did not (subgroup-B1), a gap between males and females emerged with females showing 1.55-folds higher risk than males (OR = 5.33; 2.32–12.23 and 3.44; 1.58–7.49 respectively). Due to the evident overrule of colon surgery in adhesions formation and to reveal any possible associations of AA with the other kinds of LAP, we excluded colon surgery from the computation. Nevertheless, no significant improvement in OR-values was obtained, with the only exception of hysterectomy that progressively increased OR-values from 0.63 (A vs B) up to 2.71 (A1 vs B1) after colon surgery exclusion.

**Table 3:** Risk of developing AA or bowel obstruction according to different types of original LAP.

Type of LAP (n)	Whole group of LAPs (n = 426) [♀/♂, 235/191; 55.2% ♀]						All LAPs (no colon surgery) (n = 275) [♀/♂, 168/107; 61.1% ♀]			
	A (n = 212) n (%)	A *vs* B OR (CI; 95%)	P	A1 (n = 117) n (%)	A1 *vs* B1 OR^1^ (CI; 95%)	P	A *vs* B OR (CI; 95%)	P	A1 *vs* B1 OR (CI; 95%)	P
*colon surgery (n = 151)* ♀, n = 67 ♂, n = 84	112 (74.2) 47 (70.1) 65 (77.3)	5.1 (3.24–7.8) 5.24 (2.82–9.72) 4.2 (2.22–7.95)	**0**.**0001** **0**.**0001** **0**.**0001**	69 (45.7) 28 (41.8) 41 (48.8)	4.41 (2.51–7.76) 5.33 (2.32–12.23) 3.44 (1.58–7.49)	**0**.**0001** **0**.**0001** **0**.**0001**	**—**	—	**—**	**—**
*appendectomy (n = 123)* ♀, n = 72 ♂, n = 51	43 (34.9) 22 (30.5) 21 (41.2)	0.43 (0.28–0.66) 0.49 (0.27–0.88) 0.36 (0.19–0.7)	**0**.**0001** **0**.**0150** **0**.**0022**	22 (17.9) 7 (9.7) 15 (23.4)	0.39 (0.22–0.72) 0.28 (0.11–0.73) 0.49 (0.21–1.10)	**0**.**0022** **0**.**0071** 0.08	0.9 (0.55–1.47) 0.97 (0.5–1.9) 0.75 (0.35–1.6)	n.s. n.s. n.s.	0.89 (0.44–1.8) 0.58 (0.2–1.68) 1.11 (0.40–3.06)	n.s. n.s. n.s.
*other surgery: splenectomy*, *cholecystectomy*, *(n = 93)* ♀, n = 37 ♂, n = 56	37 (39.8) 10 (27.0) 27 (48.2)	0.59 (0.37–0.95) 0.45 (0.21–0.98) 0.53 (0.28–0.99)	**0**.**029** **0**.**042** **0**.**047**	15 (16.1) 3 (8.1) 12 (21.4)	0.43 (0.22–0.86) 0.26 (0.07–0.97) 0.44 (0.19–1.05)	**0**.**0146** **0**.**0347** 0.0605	1.25 (0.74–2.10) 0.78 (0.35–1.77) 1.33 (0.62–2.86)	n.s. n.s. n.s.	0.89 (0.42–1.90) 0.5 (0.13–1.97) 0.9 (0.33–2.48)	n.s. n.s. n.s.
Hysterectomy* (n = 59)	20 (33.9)	0.63 (0.34–1.16)	n.s.	11 (18.6)	0.96 (0.4–2.34)	n.s.	1.23 (0.63–2.43)	n.s.	2.71 (0.96–7.72)	0.057

Significant P-values are shown in bold. *Only females were computed in the analysis.

### Bowel obstruction occurrence stratified by different LAP (whole survival analysis)

**Figure 3:**
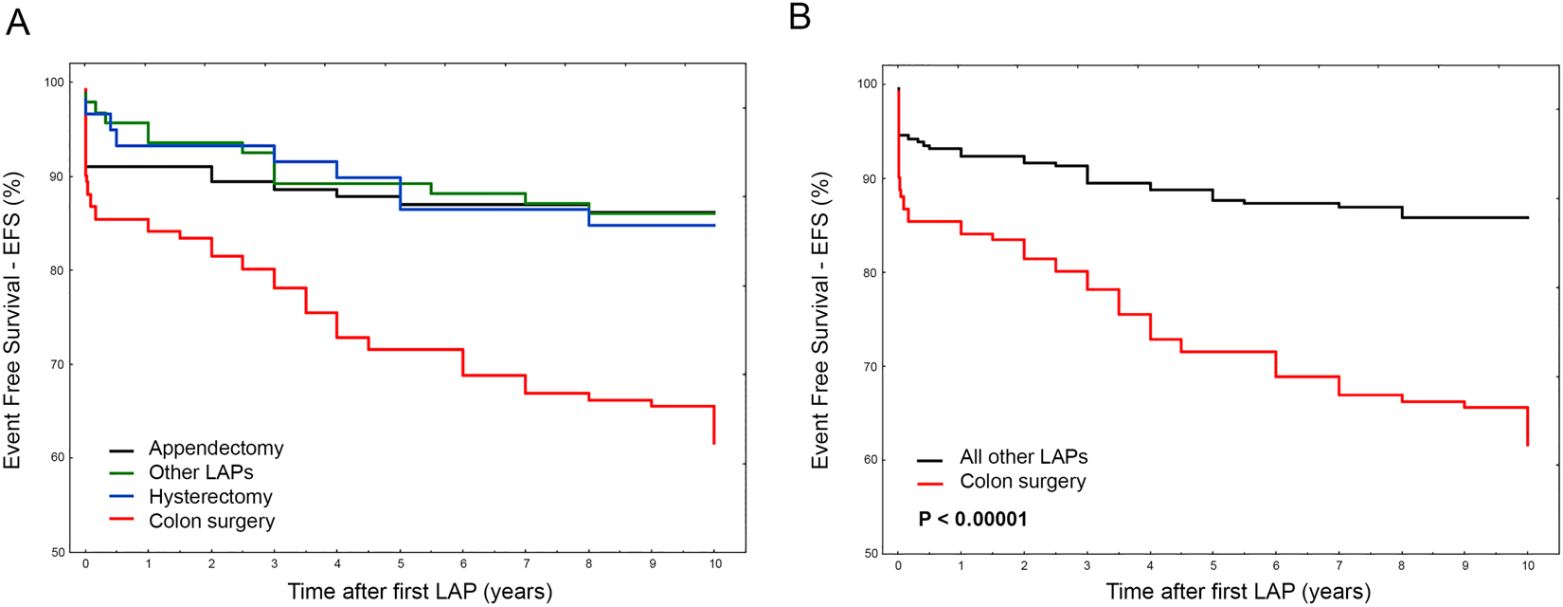
(**A**,**B**) Event Free Survival (EFS) at 10-years survey for the different type of original LAP. In (**A**) bowel obstruction occurrence was analyzed according to the different LAP in the whole cohort of patients. Colon surgery (red line) differs in survival compared to the remaining LAPs that show similar trend and frequency. In (**B**) bowel obstruction occurrence after colon surgery was compared to that obtained from the remaining LAPs taken together (i.e. appendectomy, splenectomy, cholecystectomy and hysterectomy).

By stratifying bowel obstruction rates according to the different LAP, it appears that colon surgery completely differs in survival rate both at 10- and 5-years survey, while the remaining LAPs showed similar trend and frequency (Fig. 3A). For this reason, we performed a comparison between colon surgery and the remaining combined LAPs. Taken together, appendectomy, splenectomy, cholecystectomy and hysterectomy had minor effect on EFS than colon surgery (Fig. 3B), resulting in a 3-folds higher risk to develop bowel obstruction after colon surgery than after the remaining LAPs (HR_10y_ = 3.02, 2.0–4.54; P < 0.00001 and HR_5y_ = 2.48, 1.58–3.9; P = 0.000075).

### Bowel obstruction occurrence stratified by different LAP (gender-related survival analysis)

Globally, by comparing the rate of AA occurrence in the whole cohort of cases, it stands out that males overtake females (♂ 59.1% *vs* ♀ 42.1%; P = 0.006) as well as by comparing the rate of bowel obstruction occurrence (♂ 35.6% *vs* ♀ 20.8%; P = 0.001).

**Figure 4:**
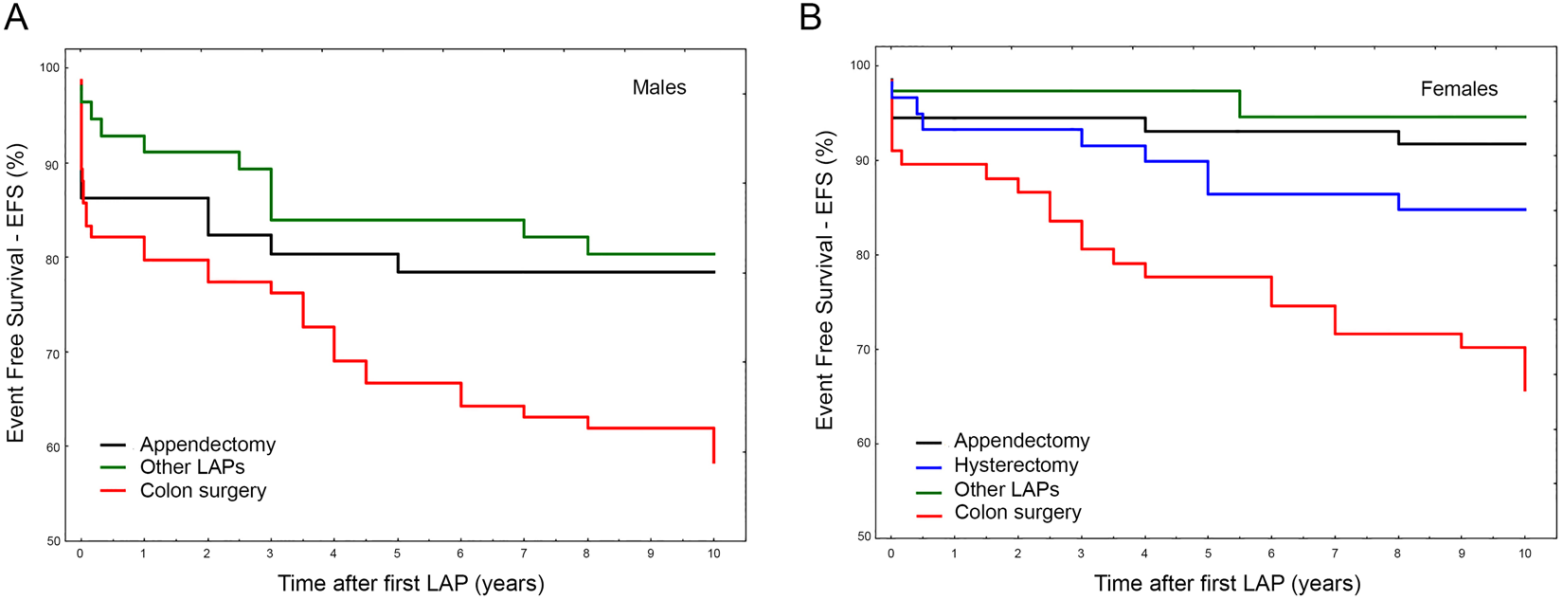
(**A**,**B**) Gender sub-analysis for bowel obstruction occurrence by different LAP. In (**A**), Event Free Survival (EFS) at 10-years survey for the different type of original LAP in males. In (**B**) Event Free Survival (EFS) at 10-years survey for the different type of original LAP in females.

**Figure 5:**
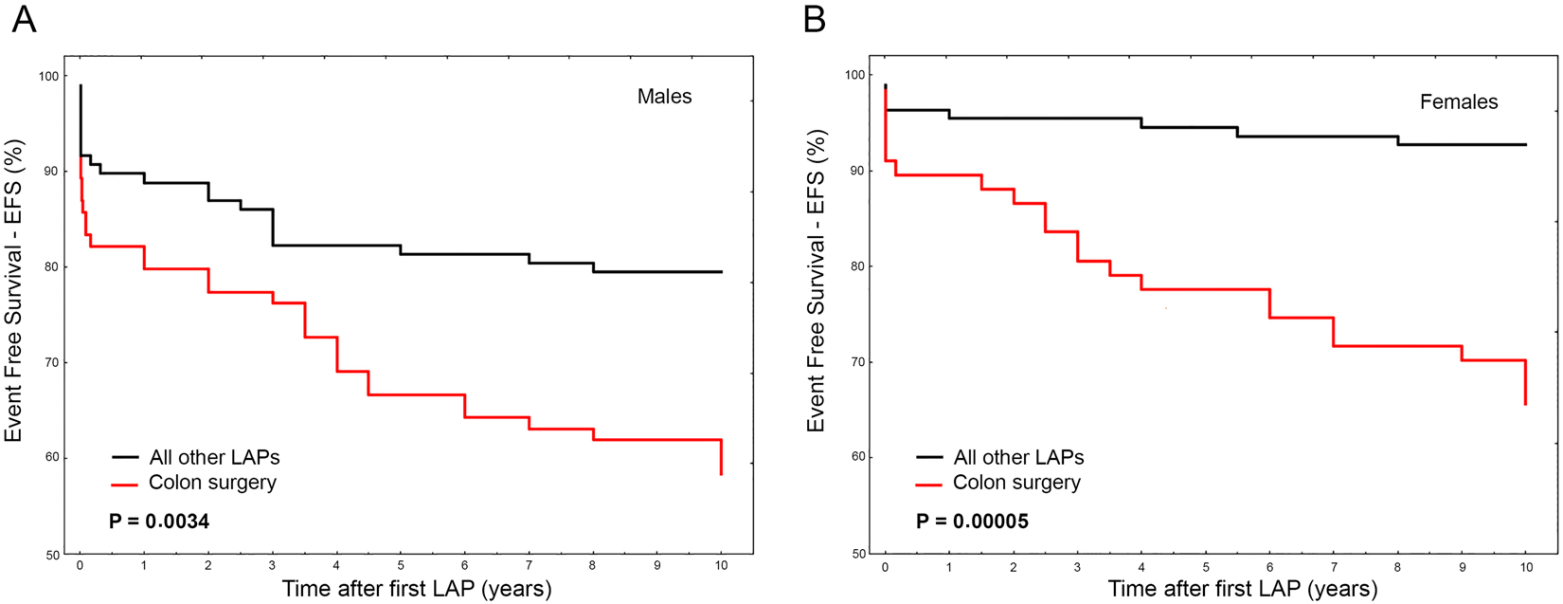
(**A**,**B**) Gender sub-analysis for bowel obstruction occurrence of colon surgery *vs* the other LAP taken together. Event Free Survival (EFS) at 10-year survey after colon surgery compared to that of the remaining LAPs taken together (i.e. appendectomy, splenectomy, cholecystectomy) in males (**A**) and females (**B**).

These data do not match with the higher ORs observed in females particularly for colon surgery (see Table 3) contrasting in turn with the EFS analyses stratified by sex ascribing to males almost double risk of bowel obstruction respect to females after any LAP (♂ *vs* ♀: HR_10y_ = 1.87, 1.24–2.8; P = 0.0024 and HR_5y_ = 2.03, 1.34–3.38; P = 0.0013). For these reasons, we performed a separate sex-specific sub-analysis stratifying bowel obstruction events by different LAP (Fig. 4A,B). As expected, colon surgery yielded the lowest survival rate and this was comparable within both sexes, thought males suffered slightly more than females (Supplementary Fig. 1). Accordingly, we merged appendectomy, splenectomy and cholecystectomy and compared them with colon surgery in a further sex sub-analysis (Fig. 5A,B). Although colon surgery equally affected females and males in terms of EFS (♀ *vs* ♂: P_10y_ = 0.317 and P_5y_ = 0.159), the other surgical procedures taken together affected more males than females (♀ *vs* ♂: P_10y_ = 0.013 and P_10y_ = 0.017). For this reason, the risk for bowel obstruction after colon surgery with respect to the other kind of surgery was almost 2.5-folds higher in females than in males (♀HR = 5.28, 2.36–11.8; P = 0.00005 and ♂HR = 2.22, 1.31–3.85; P = 0.0034). The inclusion of hysterectomy among the other surgeries in female group slightly reduced the global risk (HR = 3.85, 2.0–7.14; P = 0.000033).

### FXIII SNPs in the whole cohort of patients and in subgroups: ORs calculation

Genotype distributions and allele frequencies for *F13A1* and *F13B* genes are shown in Supplementary Table 2 for the whole cohort and the different subgroups of patients. Risk calculation by the possible genetic models in patients’ groups and subgroups are shown in Table 4. It is to note that the most consistent ORs were obtained by comparing subgroup A1 *vs* B1, i.e. those patients with AA complicated by bowel obstruction *vs* those characterized by surgically verified absence of AA. Likewise, it is also interesting the absence of any significant score by comparing subgroup A2 *vs* B1, i.e. those patients with asymptomatic AA *vs* those characterized by surgically verified absence of AA. Accordingly, Table 5 summarises the most significant and reliable ORs obtained by comparing patients characterized by different extreme clinical phenotypes. Overall, by stratifying case-patients according to the presence/absence of bowel obstruction (i.e. A1/A2) and control-patients according to the absence of AA development or symptoms (i.e. B1/B2), the frequency of FXIII polymorphic alleles increased in case-patients and decreased in control-patients (Supplementary Table 2) with expected effects on the consequent risk calculation (Table 5). In the OR calculation, this was even more evident when comparing the extreme phenotypes of cases *vs* controls (i.e. A1 *vs* B1), particularly for Y204F and P564L (Table 5, right panel). Interestingly, the intra-group comparison of patients with AA and bowel obstruction (subgroup-A1) with those with AA without bowel obstruction (subgroup-A2), yielded higher risk (e.g. PP_564_ *vs* LL_564_; OR = 10.0; 1.24–80.3; P = 0.010)_,_ strongly supporting the role of FXIII SNPs in the prediction of AA complications being worthy of dedicated survival analyses. Finally, OR-scores higher than 20-folds were occasionally detected in the analyses (Tables 4 and 5) by the use of approximation formula (see Methods) but they were reported just to guess stronger associations even though it should not be overlooked that the absence of specific genotypes among particular subgroups (i.e. LL_564_ homozygotes in B1 subgroup) might underline a biological meaning.

**Table 4:** FXIII genotype distributions among the whole cohort of cases and in the subgroups and ORs computation.

Groups	OR, 95% CI; P FXIIIA V34L				OR, 95% CI; P FXIIIA Y204F		OR, 95% CI; P FXIIIAP564L				OR, 95% CI; P FXIIIB H95R			
	VV *vs* LL	VV *vs* VL + LL	VV + VL *vs* LL	V *vs* L	YY *vs* YF	Y *vs* F	PP *vs* LL	PP *vs* PL + LL	PP + PL *vs* LL	P *vs* L	HH *vs* RR	HH *vs* HR + RR	HH + HR *vs* RR	H *vs* R
**Group A** *vs* **Group B**	1.32 (0.6–2.9) 0.474	0.96 (0.6–1.4) 0.905	1.37 (0.6–2.9) 0.420	1.03 (0.8–1.4) 0.812	5.25 (1.14–24.2) **0**.**033**	5.14 (1.12–23.6) **0**.**035**	2.43 (0.8–7.2) 0.107	1.30 (0.87–1.9) 0.196	2.29 (0.8–6.7) 0.131	1.33 (0.94–1.9) 0.103	0.15 (0.01–2.9) 0.220	1.36 (0.82–2.26) 0.20	0.14 (0.01–2.8) 0.197	1.20 (0.75–1.9) 0.445
**Group A** *vs* **Group B1**	3.22 (0.9–11.4) 0.067	1.40 (0.9–2.3) 0.151	3.02 (0.9–10.6) 0.084	1.50 (1.0–2.3) **0**.**05**	5.59 (0.7–44.3) 0.102	5.48 (0.7–43.1) 0.105	14.84 (0.86–255.1) 0.063	1.78 (1.07–2.9) **0**.**025**	13.07 (0.8–223.8) 0.076	1.87 (1.2–2.9) **0**.**006**	0.11 (0.01–2.3) 0.157	1.19 (0.66–2.2) 0.55	0.11 (0.01–2.2) 0.148	1.06 (0.61–1.8) 0.848
**Group A1** *vs* **Group B1**	3.34 (0.9–12.8) 0.078	1.45 (0.8–2.5) 0.174	3.08 (0.8–11.7) 0.097	1.52 (0.97–2.4) 0.070	8.29 (1.02–67.4) **0**.**047**	8.04 (1.0–64.8) **0**.**05**	27.00 (1.55–469.1) **0**.**023**	2.26 (1.3–3.96) **0**.**004**	22.37 (1.3–386.4) **0**.**032**	2.42 (1.5–3.9) **0**.**0004**	0.21 (0.01–4.5) 0.320	1.57 (0.82–3.0) 0.172	0.19 (0.01–4.0) 0.287	1.34 (0.74–2.4) 0.337
**Group A1** *vs* **Group B**	1.37 (0.5–3.4) 0.497	1.00 (0.6–1.6) 0.997	1.40 (0.6–3.4) 0.458	1.06 (0.7–1.5) 0.763	7.78 (1.62–37.3) **0**.**010**	7.54 (1.6–35.8) **0**.**011**	4.42 (1.45–13.4) **0**.**008**	1.66 (1.04–2.6) **0**.**032**	3.91 (1.3–11.7) **0**.**015**	1.71 (1.2–2.5) **0**.**006**	0.29 (0.01–5.0) 0.416	1.8 (1.01–3.15) **0**.**044**	0.26 (0.03–5.0) 0.370	1.53 (0.9–2.5) 0.114
**Group A2** *vs* **Group B1**	3.08 (0.8–12.4) 0.108	1.32 (0.7–2.3) 0.266	2.44 (0.7–11.7) 0.125	1.41 (0.9–2.4) 0.123	2.43 (0.2–27.2) 0.471	2.41 (0.2–26.8) 0.473	3.86 (0.15–96.2) 0.410	1.29 (0.7–2.4) 0.413	3.63 (0.15–90.2) 0.431	1.29 (0.74–2.2) 0.370	0.23 (0.01–4.9) 0.347	0.8 (0.37–1.7) 0.551	0.24 (0.01–4.9) 0.352	0.72 (0.35–1.5) 0.370

Where zeros cause problems with computation of the odds ratio or its standard error, 0.5 is added to all cells (Pagano & GauVreau, 2000; Deeks & Higgins, 2010). https://www.medcalc.org/calc/odds_ratio.php. Significant P-values are shown in bold.

**Table 5:** Selected OR computations in the whole cohort of cases and in the subgroups.

Clinical Phenotype *(Group)*		OR, 95% CI; P				Clinical Phenotype *(Group)*		OR, 95% CI; P			
		FXIIIA V34L	FXIIIA Y204F	FXIIIA P564L	FXIIIB H95R			FXIIIA V34L	FXIIIA Y204F	FXIIIA P564L	FXIIIB H95R
Presence of AA *(A; n* = *212)*	Absence of AA or symptoms *(B; n* = *214)*	1.03 (0.8–1.4) 0.812	5.14 (1.12–23.6) **0**.**035**	2.43* (0.82–7.2) 0.107	1.36^§^ (0.82–2.26) 0.20	Presence of AA and bowel obstruction *(A1; n* = *117)*	Absence of AA or symptoms *(B; n* = *214)*	1.06 (0.7–1.5) 0.763	7.54 (1.6–35.8) **0**.**011**	4.42* (1.45–13.4) **0**.**008**	1.8^§^ (1.01–3.15) **0**.**044**
	Absence of AA at LAP *(B1; n* = *114)*	1.50 (1.0–2.3) **0**.**05**	5.48 (0.7–43.1) 0.105	14.84* (0.86–255.1) 0.063	1.19^§^ (0.66–2.2) 0.55		Absence of AA at LAP *(B1; n* = *114)*	1.52 (0.97–2.4) 0.070	8.04 (1.0–64.8) **0**.**05**	27.00* (1.55–469.1) **0**.**023**	1.57^§^ (0.82–3.0) 0.172

All the OR calculations were obtained by allele frequencies comparisons with the exception of ORs calculated by comparing the opposite homozygous conditions^*^ and ORs calculated by the genetic dominant model^§^. Only the most relevant ORs are shown. The complete list of ORs is shown in Supplementary Table 2. Significant P-values are shown in bold.

### FXIII SNPs survival analyses in the whole cohort and in subgroups: single analyses

**Figure 6:**
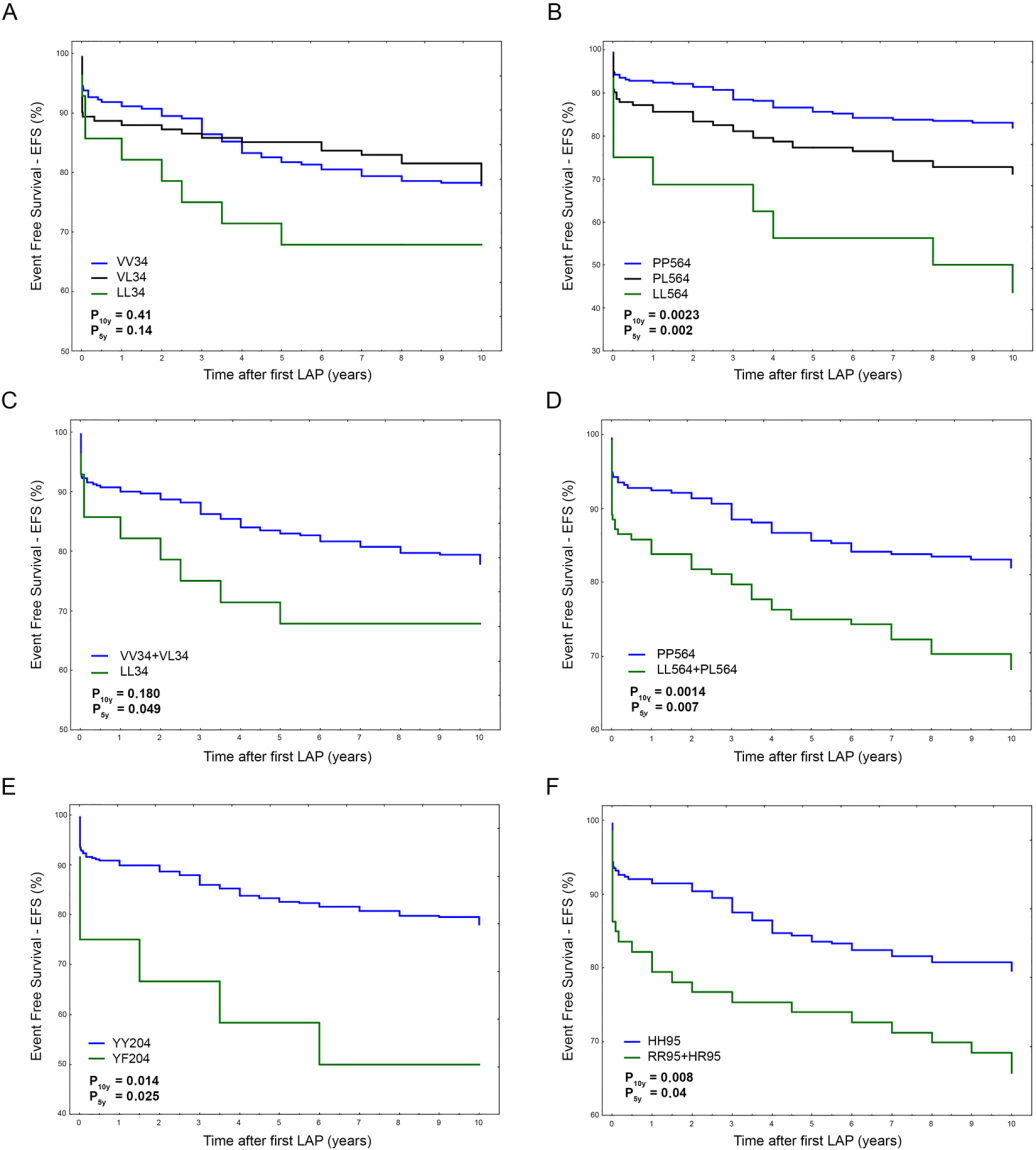
(**A–F**) Overall Event Free Survival (EFS) for bowel obstruction occurrence at 10-years survey according to different FXIII SNPs considered. *F13A1* V34L (VV *vs* VL *vs* LL) (**A**); *F13A1* P564L (PP *vs* PL *vs* LL) (**B**); *F13A1* V34L (VV + VL *vs* LL) (**C**); *F13A1* P564L (PP *vs* PL + LL) (**D**); *F13A1* Y204F (YY *vs* YF) (**E**); *F13B* H95R (HH *vs* HR + RR) (**F**).

Considering that bowel obstruction due to AA can occur also many years after the original tissue injury, we aimed to identify possible time-dependent associations of FXIII genes with bowel obstruction. We therefore stratified patients by the different FXIII SNPs and as before, we performed short and extended survival analyses from the first original LAP (5- and 10-years respectively). Interestingly, V34L gene variant followed a genetic recessive model, differently from P564L that showed a gene-dosage-effect in line with a dominant genetic model (Fig. 6A,B). For this reason, we computed VV_34_ + VL_34_ *vs* LL_34_ and PP_564_ *vs* PL_564_ + LL_564_ in survival analysis and hazard risk calculations (Fig. 6C,D). The two other SNPs (i.e. Y204F and H95R) did not include (enough) homozygous cases for the polymorphic rare allele (FF_204_ and RR_95_ respectively) so we performed a classical carrier *vs* no-carrier two-group comparison (Fig. 6E,F).

### FXIII SNPs survival analyses in the whole cohort and in subgroups: combined analyses

Though at different extent, the presence of any polymorphic allele in the FXIII gene was associated to increased risk to develop bowel obstruction both at longer and shorter follow-up (Table 6). Therefore, we compared those patients wild-type for each considered SNP (common alleles) with those carrying at least one polymorphic allele (rare alleles) in any FXIII gene. As shown in Table 6, no changes in the risk score was obtained by merging all carriers together (any SNP) and the resulting hazard risks were in the range of those obtained by single SNP analyses. Accordingly, the scores for the comparison of the whole groups (A *vs* B) were HR_10y_ = 2.49, 1.61–3.84 (P = 0.00004) and HR_5y_ = 2.16, 1.34–3.50 (P = 0.0015) for extended and short survival respectively and the subgroup comparison (A1 *vs* B1) did not modify the risk calculation.

**Table 6:** Risk for bowel obstruction occurrence at different follow-up in the whole cohort and sub-groups.

FXIII genotypes	A1 *vs* B1		A *vs* B	
	10 years survival HR, 95% CI; P	5 years survival HR, 95% CI; P	10 years survival HR, 95% CI; P	5 years survival HR, 95% CI; P
FXIIIA V34L	2.69 (1.35–5.36) **0**.**005**	3.1 (1.54–6.23) **0**.**0015**	1.6 (0.8–3.15) 0.180	2.00 (1.00–4.02) **0**.**049**
FXIIIA P564L	2.12 (1.43–3.17) **0**.**0002**	1.99 (1.28–3.13) **0**.**0025**	1.91 (1.28–2.85) **0**.**0014**	1.85 (1.18–2.89) **0**.**007**
FXIIIA Y204F	2.11 (0.92–4.83) 0.076	2.07 (0.34–5.14) 0.11	2.81 (1.23–6.4) **0**.**014**	2.81 (1.13–6.95) **0**.**025**
FXIIIB H95R	1.53 (0.97–2.42) 0.065	1.43 (0.85–2.4) 0.176	1.84 (1.17–2.9) **0**.**008**	1.71 (1.02–2.88) **0**.**04**
Any SNP	2.49 (1.60–3.85) **0**.**00004**	2.11 (1.31–3.41) **0**.**002**	2.49 (1.61–3.84) **0**.**00004**	2.16 (1.34–3.50) **0**.**0015**

Significant P-values are shown in bold.

**Figure 7:**
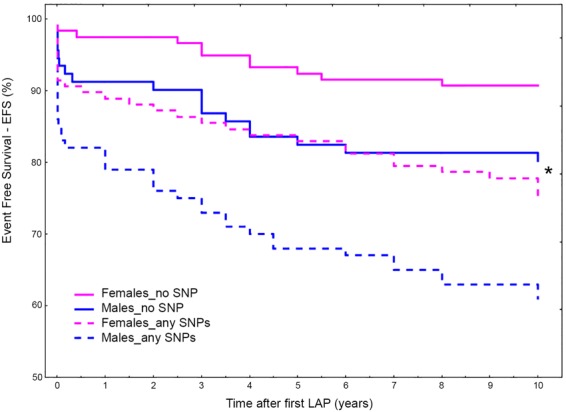
Overall Event Free Survival (EFS) for bowel obstruction occurrence at 10-years survey for FXIII SNPs (any) in male and females. EFS in patients carrying any of the considered SNPs *vs* patients carrying no-SNPs (i.e. wild type for all SNPs considered). (*) Interestingly, females carrying any SNPs (dashed pink line) and males wild types for all the SNPs considered (solid blue line) show completely overlapping survival curves.

Finally, also the gender specific analysis confirmed the role of genetic risk-factor to the presence of any FXIII SNP in the genotype of patients, ascribing to FXIII A and B gene the function of inherited predisposition to AA formation or bowel obstruction occurrence after surgery. In detail, even though males and females had similar HRs (♀HR = 2.88, 1.44–5.76; P = 0.0028 and ♂HR = 2.21, 1.26–3.87; P = 0.0054), there were evident differences in their trends (Fig. 7) so that, in a mutual comparison, the presence of any FXIII SNPs ascribed to males 1.5-folds higher risk than females (HR = 1.51; 1.04–2.19; P = 0.032). Interestingly, males wild type for the SNPs investigated had EFS overlapping that of female carriers (Fig. 7).

## Discussion

Despite strong evidence that AA are the most common complication in abdominal surgery, little progress has been made in the recognition of informative molecular markers to predict AA or bowel obstruction occurrence^2^. Unlike other types of postoperative complications, AA make patients at lifelong risk for various pathologic conditions. For this reason, recognition of predictive inherited predispositions and/or biomarkers useful to recognize in advance those at risk patients could help clinicians in establishing prevention targeted programs. In Europe and North America, large part of general surgery elective procedures is reoperation because of AA. This leads to high related costs in both sexes, although it should be taken into account that expenses concerning fertile-females are more than 2-folds higher if considering the elevated tubal infertility rate due to AA requiring costly *in vitro* fertilization IVF-procedures^30,31^. To contrast AA formation and the subsequent economic burden for the national health care systems, cost-benefits of anti-AA barrier trials have been performed. These gave encouraging results, particularly for reducing those complications requiring reoperation, though data on preservation of female fertility are not definitive^4,31–34^. Placing anti-AA barriers is costly and needs extra operative time, therefore barriers positioning should be preferentially targeted in selected high-risk cases^35^. Patients with AA and chronic abdominal pain have been approached with laparoscopic adhesiolysis but the procedure is still controversial, often causing other AA in a vicious circle and it is not clear if adhesiolysis leads to a substantial pain relief, preservation of female fertility or improvement in quality of life^36,37^.

Together with the expected different degree in the risk score ascribable to the different kinds of LAP, the major finding of the present study is that the main SNPs within *F13A1* or *F13B* genes have a role of inherited molecular markers in predicting AA and/or bowel obstruction with interesting gender distinctions.

Accordingly, among colon surgery intervention, at least 75% of patients developed AA and among these, more than 60% experienced bowel obstruction. On the contrary, the remaining LAPs yielded a lower rate overall below 20%. In a gender perspective, despite the evidence that males more frequently experienced AA or bowel obstruction than females, the latter seemed to have higher risk scores after colon surgery. The apparent conflicting result can be explained by the observation that, while colon surgery gave comparable rates of bowel obstruction in both sexes, females less frequently experienced this complication after any of the remaining LAPs, affecting in turn the intra-gender risk calculation. As expected, the gender-specific sub-analysis ascribed to colon surgery the strongest trigger for AA or bowel occlusion in both sexes but females suffered more colon surgery in respect to the remaining other LAPs and this ascribed them an higher risk (♀HR = 5.28, 2.36–11.8; P = 0.00005 and ♂HR = 2.22, 1.31–3.85; P = 0.0034).

The genetic analyses produced interesting results, particularly with regard to the identification of early prognostic molecular markers as risk indicators useful in the clinical practise. In detail, FXIIIA P564L and FXIIIA Y204F showed the highest scores, with crude ORs ranging from 4.42- (PP *vs* LL) up to 8.29-folds (YY *vs* YF) in group-A1 *vs* group-B and group-A1 *vs* group-B1 respectively. Considering that group-A1 included the worst extreme clinical phenotypes, such association could ascribe to the SNPs a potential role as prognostic markers for bowel obstruction after surgery. In addition, the comparison between patients with AA in the absence of bowel obstruction with patients who did not develop AA (group A2 *vs* B1), did not yield any significant association suggesting that FXIII SNPs could actually predict the progression from asymptomatic AA to the severest clinical phenotype. Accordingly, the further intra group analysis was performed among all the patients who developed AA, i.e. those with- *vs* those without- bowel obstruction (groups A1 *vs* A2). This analysis definitely confirmed our hypothesis, yielding a significant ten-fold increased risk of bowel obstruction when the opposite homozygotes were compared (PP vs LL). Of interest, also the comparison of the whole groups (A *vs* B) gave significant results with more than five-fold increased risk in patients carrying Y204F variant, even though it should be considered that these two groups also include cases with AA in absence of bowel obstruction over a period of time of almost ten years (subgroup-A2) and cases that merely did not reported symptoms related to AA after the first LAP (subgroup-B2). This observation contrasts in part with our previous speculation about the potential role of FXIII genes as prognostic indicators of bowel obstruction. Actually, it is impossible to predict “if” and “when” bowel obstruction develops in a patient presenting AA, due to the multifactorial nature of this severe complication. Therefore, we could only speculate to consider FXIII genes as predictors of both AA and their complications in surgical patients.

Summarizing, given that all the cases enrolled in our study underwent to previous LAP and:

- or they developed bowel obstruction within 10-years after the first LAP (A1);
- or they did not develop bowel obstruction in a mean period of about 10-years in spite of AA (A2);
- or they did not develop bowel obstruction and AA in a period of at least 10-years (B1);
- or they did not have symptoms related to AA presence for a period of at least 10-years (B2);

we considered extremely interesting to investigate if those FXIII SNPs associated with AA or bowel obstruction also had effects on the EFS by dedicated Kaplan-Meier analyses.

Of interest, though at different extent, the presence of any SNPs in the FXIII genes significantly affected EFS, yielding HRs of about 2.5-folds at 10-years follow-up (P = 0.00004). In detail, FXIIIA V34L reached the highest HR up to 3.1-folds at 5-years follow-up and in general the extreme phenotype comparison (i.e. A1 *vs* B1) overall gave higher risk scores. This was particularly evident for the more common V34L and P564L variants with respect to the less frequent polymorphisms (i.e. Y204F and H95R) in which groups’ sub-setting affected to a major extent the statistical power.

Additionally, by means of gender analysis we assessed if FXIII genes had different ability in predicting bowel obstruction. Even though the intra-group analyses gave similar risks for males and females, the inter-groups analyses ascribed to the presence of any SNP a risk score summed-up as follows: any-SNP♂ > any-SNP♀ ≅ no-SNP♂ > no-SNP♀. This resulted in the paradox that females carrying any-SNP showed a risk as low as that of males carrying no-SNPs.

These data suggest that males and females experienced the risk for abdominal post surgery complications in a completely different way, both considering the primary risk due to surgery and the inherited genetic risk predisposition.

Postsurgical AA formation begins with injury to the mesothelial surfaces, leading to the exposure of the basal membrane and activation of the several molecular processes aimed at repairing the damage. Among the compounds effective in preventing AA, some drugs belong to the anticoagulant/fibrinolysis area, presumably by inhibiting platelets and excessive fibrin deposition, while others belonging to the anti-inflammatory area modulate neutrophils/macrophage response, cytokine cascade and vascular permeability^4^.

FXIII stays at the intersection of coagulation, fibrinolysis, inflammation and infection control^12–14^ all key steps that, when unbalanced, are responsible for abnormal wound-healing that is crucial for AA formation. After injury, FXIII finely tunes fibrin-meshwork during blood coagulation by forming the best substrate and environment, giving a provisional 3D-scaffold for successive cell growth and ECM protein deposition. Failure of the fibrinolytic system or delayed break-down of fibrin meshwork might result in unnecessary scaffold perpetuation into which fibroblasts and other reparative cells can migrate and proliferate with excessive deposition of collagen and other ECM proteins, supporting a permanent fibrous connective tissue.

The main investigated V34L SNP accounts for an earlier release of the activation peptide of FXIIIA, being very close to the thrombin-activation-site (R37-G38), whereas the H95R supports the FXIIIA2B2 tetramer dissociation essential for complete FXIII activation. V34L SNP is considered the main functional locus among FXIII SNPs^38^ whilst the remaining SNPs have been less investigated, though they have gained wide attention in the field of thrombosis and wound healing because of effects on both FXIII level and activity^21–25,39^. Consequently, a gene variant might act as a protective inherited predisposition when it counteracts a delayed or inefficient healing as the case of chronic skin lesions^21–24,40–44^. Conversely, the same gene variant, when set in an unrestrained or dysregulated process, might lead to excessive repairing with negative consequences and complications such as fibrosis and AA establishment as previously described in different contexts of other complex and multifactorial diseases^45–47^.

After tissue injury, multi-factorial and complex mechanisms of reparative processes start where different genes play a role. Although inflammation, angiogenesis and immunity are key players, reparative processes primarily involve coagulation and fibrinolysis. As a consequence adequate 3D-fibrin scaffold organization might be responsible for optimal healing (no adhesions) whilst unbalanced or excessive healing might favour adhesions. For these reasons we looked at coagulation FXIII gene necessarily involved in any healing process. In addition, further candidate genes including *IL-IRN*, *PAI-1*, *FGB*, *TAFI* and *HLA* have been considered as emerging factors in post-operative adhesions pathogenesis^4,26–29^.

Nowadays, the use of molecular markers predicting risk and progression of a disease is a practical and handily tool, successfully translated in the clinical practice at affordable cost with advantageous cost/benefit ratio. The recognition of predictive inherited predispositions or biomarkers useful in scoring in advance at risk patients could avoid waste of money and contrast the economic burden for national health care systems by targeting AA-barriers positioning in selected high-risk patients. To optimize the clinical exploiting of this approach, the development of dedicated-customized SNPs-micro-arrays could further reduce the costs and open the way for feasible and cost-effective precision medicine procedures.

Our results show that inherited genetic predispositions favour the development of abdominal post-surgical complications in a gender-specific fashion. Confirmation studies in larger cohorts and multicentre studies will validate their strong pharmacogenetics role in a translational and precision medicine perspective.

## Material and Methods

### Patients’ selection

The study included a total of 426 patients who underwent to different kinds of medial laparotomy (LAP) at the Surgery Department of the Hospital-University of Ferrara, Italy in the period from January 2007 to December 2008. The study was performed in accordance with the Declaration of Helsinki and under the approval of the Local Ethic Committee of Ferrara, signed informed consent was obtained from all patients. In detail, they were colon surgery (n = 151; ♀, n = 67 and ♂, n = 84); appendectomy (n = 123; ♀, n = 72 and ♂, n = 51); other surgery (splenectomy and cholecystectomy n = 93; ♀, n = 37 and ♂, n = 56); hysterectomy (n = 59). We aimed at including selected patients who developed or not AA or reliable symptoms (i.e. bowel obstruction or abdominal pain) in a period of 10-years after the first LAP, unless they were hospitalised before due to suspected bowel obstruction or in occasion of a secondary independent surgical intervention where the presence of AA was eventually discovered. AA in the second LAP were scored according to the classification of the Adhesion Scoring Group^48^. In addition, we excluded all those cases in which the surgeon could not score adhesions. Finally, the clinical assessment of patients without any complication after the previous LAP included the followings: patient’s history, physical exam of the abdomen including quality of scar, presence of incisional hernia, and assessment of peristalsis in the different abdominal quadrants by ultrasound examination (Esaote My Lab 70, 3.5 MHz Convex probe, Genoa, Italy).

None of the enrolled patients was treated by anti-adhesion barriers in any forms (i.e. solid membranes, gels, liquid or spray) with the only procedure applied being extensive washing with saline-physiological solution. For this study, the selected patients were recalled for a follow-up visit and a physician informed the patient on the aims and the modality of the present study, releasing an informed consent. Blood drawing was performed only after informed consent signature and anonymized in order to perform blind genotype analyses.

The demographic and clinical characteristics of patients are shown in Table 1.

Patients’ cohort was divided as follow:

- Group A (n = 212) included patients with AA. Among these, 117 developed AA within 10 years after the first LAP diagnosed in acute because of bowel obstruction complications (subgroup-A1) and 95 had uncomplicated AA occasionally detected during a second LAP performed in elective surgery without limit of time from the original LAP (subgroup-A2).
- Group B (n = 214) included 114 patients without AA confirmed during a second LAP performed in election at least 10 years after the original LAP (subgroup-B1) and 100 patients who did not have symptoms of abdominal pain and colic and/or digestive disorders following the first abdominal procedure for a period of at least 10 years (subgroup-B2).

Exclusion criteria were presence in patients of cancer, autoimmune diseases, post-surgery infective complication or any other cause affecting normal post-surgery healing.

### Genotyping

Venous whole blood was collected from patients by venipuncture into Vacutainer tubes with sodium citrate and immediately frozen at −80 °C. DNA extraction and purification (BioRobot EZ1 system QIAGEN; Hilden, Germany), PCR-amplification (Sure Cycler 8800; Agilent Technologies) and genotyping by Pyrosequencing (PyroMark Q96 ID, Qiagen) were performed according to our previous reports^21–24^. The list of primers used to amplify/sequence the target genes specific for FXIIIA V34L, FXIIIA P564L, FXIIIA Y204F, and FXIIIB H95R SNPs is shown in Supplementary Table 1. All the oligo-sequences of the investigated SNPs (Forward, Reverse and Sequence primers) were selected to have at least 98.0% compatibility score. According to our internal quality control procedures^23,45^, we confirmed haplotypes by re-genotyping about 20% of randomly selected samples among each different genotype for each specific SNP by enzymatic restriction analyses^24,39^. There were no discrepancies between genotypes detected in duplicate and/or by different methods. Known genotypes were used as internal control references.

### Statistical analysis

Statistical differences among groups were assessed by Chi-squared test and Student’s t-test for genotype distribution and mean ± SD value comparisons respectively. Yates’ correction or Fisher’s exact test were applied when appropriate. Odds Ratio (OR) and 95% confidence interval (95% CI) were used to estimate the risk associated to different LAP or SNPs. ORs and P-values were calculated comparing subgroups by all the genetic models including allelic distribution. Where zeros cause problems with computation of the odds ratio (OR) or standard error, approximation formula was used by adding 0.5 to each cell in the contingency table (https://www.medcalc.org/calc/odds_ratio.php) as previously reported^45^. Survival curves were constructed by the Kaplan-Meier method and event free survival (EFS) among groups was compared using the Log-Rank test. To estimate the risk of having a poor clinical outcome in terms of AA or bowel obstruction, hazard risk (HR) and 95% CI were calculated between different LAP and classes of genotypes by means of Cox-proportionate hazards modelling. P-values ≤ 0.05 were considered significant. Deviation from Hardy-Weinberg equilibrium was calculated for each polymorphism in groups and subgroups. Analyses were performed by using SPSS Statistical Package (Version 22; SPSS Inc., Chicago, IL, USA) and Statistica software (Version 13.3).

## Electronic supplementary material

Supplementary material
