# Supplementary material for: Inherited genetic predispositions in F13A1 and F13B genes predict abdominal adhesion formation: identification of gender prognostic indicators
Source: Sci Rep. 2018 Nov 16;8:16916. doi: 10.1038/s41598-018-35185-x (PMC6240050; doi:10.1038/s41598-018-35185-x)
Supplement: Supplementary file 1 — Supplementary material [file 41598_2018_35185_MOESM1_ESM.pdf]

Inherited genetic predispositions in *F13A1* and *F13B* genes predict abdominal adhesion formation: identification of gender prognostic indicators

Donato Gemmati[1,4*], Savino Occhionorelli[2], Veronica Tisato[3], Marco Vigliano[1], Giovanna Longo[1], Arianna Gonelli[3], Maria G. Sibilla[2], Maria L. Serino[1], Paolo Zamboni[2]

Running title: FXIII SNPs and abdominal adhesions

[1]Department of Biomedical & Specialty Surgical Sciences and Centre Haemostasis & Thrombosis, Section of Medical Biochemistry, Molecular Biology & Genetics, University of Ferrara, corso Giovecca 203, 44121 Ferrara, Italy;

[2]Department of Morphology, Surgery & Experimental Medicine, University of Ferrara and Vascular Diseases Centre, Unit of Translational Surgery, University-Hospital of Ferrara, via Aldo Moro 8, 44124 Cona-Ferrara, Italy;

[3]Department of Morphology, Surgery & Experimental Medicine and LTTA Centre, University of Ferrara, via Fossato di Mortara 70, 44121 Ferrara, Italy;

[4]University Center for Studies on Gender Medicine, University of Ferrara, 44121 Ferrara, Italy

[*]**Corresponding author**: Prof. Donato Gemmati, Department of Biomedical & Specialty Surgical Sciences, Centre Haemostasis & Thrombosis, and Center of Gender Medicine, Section of Medical Biochemistry, Molecular Biology & Genetics, University of Ferrara, corso Giovecca 203, 44121 Ferrara Italy.

Tel: (+39) 0532.237291; Fax: (+39) 0532.237291; email: d.gemmati@unife.it

Supplementary informations

Supplementary Table 1. Primer sequences for SNPs detection

Gene	dbSNP (rs)	Functional variation (a.a. change)	Oligo-primer	Oligo sequence
F13A1 **(MIM 134570)**	rs5985	V34L (Val34Leu)	Fw	5’-AATGCAGCGGAAGATGACC-3’
			Rv	5’-GCTCATACCTTGCAGGTTGAC-3’[Bio]
			Sq	5’-CACAGTGGAGTCTCAG-3’
	rs3024477	Y204F (Tyr204Phe)	Fw	5’-TGGTGTGAAGATGATGCTGTGTA-3’
			Rv	5’-TCCATAAAAAATTACCCCGAT-3’[Bio]
			Sq	5’-TGAGAAAGAAAGAGAAGAGT-3’
	rs5982	P564L (Pro564Leu)	Fw	5’-CACAACCGTTACACCATCACA-3’[Bio]
			Rv	5’-GCGTCACGTCGAACGTCT-3’
			Sq	5’-CCTTCTTGAATTCTGCC-3’
F13B **(MIM 134580)**	rs6003	H95R (His95Arg)	Fw	5’-AAAATGCACTAAGCCTGACCTGA-3’[Bio]
			Rv	5’-TCCTTCCCTCCAGTGGTTTTGTAC-3’
			Sq	5’-TGAAGCGCAACCATA-3’

Fw, Rv, Sq, indicate the Pyrosequencing forward, reverse and sequence primer respectively; [Bio], indicates biotinylated primer; *MIM*, indicates Mendelian Inheritance in Man number.

Supplementary Table 2. FXIII genotypes distributions among the whole cohort of cases and in the subgroups

**Patients, whole cohort / subgroups**	**FXIIIA V34L**			**FXIIIA Y204F**		**FXIIIAP564L**			**FXIIIB H95R**		
	**VV**<br>***n, %***	**VL**<br>***n, %***	**LL**<br>***n, %***	**YY**<br>***n, %***	**YF**<br>***n, %***	**PP**<br>***n, %***	**PL**<br>***n, %***	**LL**<br>***n, %***	**HH**<br>***n, %***	**HR**<br>***n, %***	**RR**<br>***n, %***
**Group A (n=212)**	129<br>*60.85%*	67<br>*31.60%*	16<br>*7.55%*	202<br>*95.3%*	10<br>*4.7%*	132<br>*62.26%*	69<br>*32.55%*	11<br>*5.18%*	171<br>*80.66%*	41<br>*19.34%*	0<br>*0%*
allele frequency (n=424)	***V**, n= 325*<br>*76.65%*		***L**, n= 99,*<br>*23.35%*	***Y**, n= 414*<br>*97.6%*	***F**, n= 10*<br>*2.4%*	***P**, n= 333*<br>*78.5%*		***L**, n= 91*<br>*21.5%*	***H**, n= 383*<br>*90.3%*		***R**, n= 41*<br>*9.7%*
**Group A1 (n=117)**	70<br>*59.8%*	38<br>*32.5%*	9<br>*7.7%*	109<br>*93.2%*	8<br>*6.8%*	66<br>*56.4%*	41<br>*35%*	10<br>*8.6%*	89<br>*76.1%*	28<br>*23.9%*	0<br>*0%*
allele frequency (n=234)	***V**, n= 178*<br>*76.1%*		***L**, n= 56*<br>*23.9%*	***Y**, n= 226*<br>*96.6%*	***F**, n= 8*<br>*3.4%*	***P**, n= 173*<br>*73.9%*		***L**, n= 61*<br>*26.1%*	***H**, n= 206*<br>*88%*		***R**, n= 28*<br>*12%*
**Group A2 (n= 95)**	59<br>*62.1%*	29<br>*30.53%*	7<br>*7.4%*	93<br>*97.9%*	2<br>*2.1%*	66<br>*69.5%*	28<br>*29.5%*	1<br>*1%*	82<br>*86.3%*	13<br>*13.7%*	0<br>*0%*
allele frequency (n=190)	***V**, n= 147*<br>*77.35%*		***L**, n= 43*<br>*22.60%*	***Y**, n= 188*<br>*98.9%*	***F**, n=2*<br>*1.1%*	***P**, n= 160*<br>*84.2%*		***L**, n= 30*<br>*15.8%*	***H**, n= 177*<br>*93.2%*		***R**, n= 13*<br>*6.8%*
**Group B (n=214)**	128<br>*59.8%*	74<br>*34.6%*	12<br>*5.6%*	212<br>*99%*	2<br>*1%*	146<br>*68.2%*	63<br>*29.4%*	5<br>*2.3%*	182<br>*85%*	29<br>*13.6%*	3<br>*1.4%*
allele frequency (n=428)	***V**, n= 330*<br>*77.1%*		***L**, n= 98,*<br>*22.9%*	*Y, n= 426*<br>*99.5%*	***F**, n= 2*<br>*0.5%*	***P**, n= 355*<br>*82.9%*		***L**, n= 73*<br>*17.1%*	*H, n= 393*<br>*91.8%*		***R**, n= 35*<br>*8.2%*
**Group B1 (n=114)**	78<br>*68.4%*	33<br>*28.9%*	3<br>*2.7%*	113<br>*99.1%*	1<br>*0.9%*	85<br>*74.6%*	29<br>*25.4%*	0<br>*0%*	95<br>*83.3%*	17<br>*14.9%*	2<br>*1.7%*
allele frequency (n=228)	***V**, n= 189*<br>*82.9%*		***L**, n= 39*<br>*17.1%*	***Y**, n= 227*<br>*99.6%*	***F**, n= 1*<br>*0.4%*	***P**, n= 199*<br>*87.3%*		***L**, n= 29*<br>*12.7%*	***H**, n= 207*<br>*90.8%*		***R**, n= 21*<br>*9.2%*
**Group B2 (n=100)**	50<br>*50%*	41<br>*41%*	9<br>*9%*	99<br>*99%*	1<br>*1%*	61<br>*61%*	34<br>*34%*	5<br>*5%*	87<br>*87%*	12<br>*12%*	1<br>*1%*
allele frequency (n=200)	***V**, n= 141*<br>*70.5%*		***L**, n= 59*<br>*29.5%*	*Y, n= 199*<br>*99.5%*	***F**, n= 1*<br>*0.5%*	***P**, n= 156*<br>*78%*		***L**, n= 44*<br>*22%*	***H**, n= 186*<br>*93%*		***R**, n= 14*<br>*7%*
**Total cases (n=426)**	257<br>60.33%	141<br>33.1%	28<br>6.6%	414<br>97.2%	12<br>2.8%	278<br>65.25%	132<br>30.98%	16<br>3.75%	353<br>82.86%	70<br>16.4%	3<br>0.7%
allele frequency (n=852)	***V**, n= 655*<br>*76.88%*		***L**, n= 197*<br>*23.12%*	***Y**, n=840*<br>*98.6%*	***F**, n= 12*<br>*1.4%*	*P, n= 688*<br>*80.75%*		***L**, n= 164*<br>*19.25 %*	***H**, n= 776*<br>*91.08%*		***R**, n= 76*<br>*8.92%*

Supplementary Figure 1

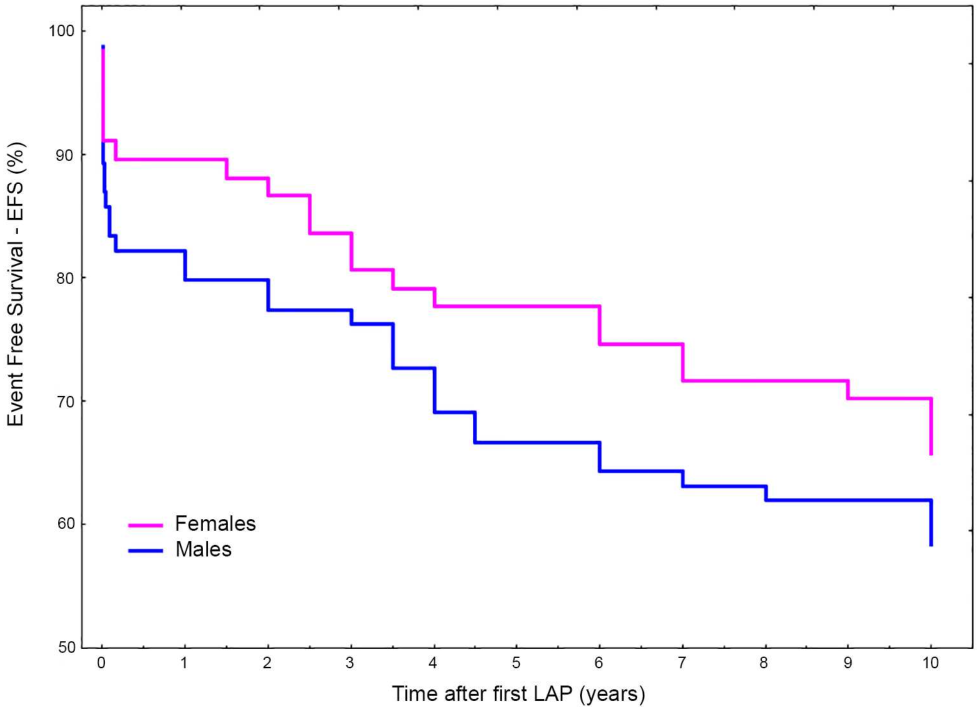

Supplementary Figure 1. Gender sub-analysis for bowel obstruction occurrence at 10-years survey after colon surgery. Colon surgery similarly affected females and males in terms of EFS; no significant difference was obtained.
